# Randomized controlled trial of tailored audit with feedback in VHA long-term care settings

**DOI:** 10.1186/s43058-023-00510-7

**Published:** 2023-10-26

**Authors:** Jennifer Kononowech, Winifred Scott, Zach Landis-Lewis, Anne E. Sales

**Affiliations:** 1 Center for Clinical Management Research, VA Ann Arbor Healthcare System, 2800 Plymouth Rd, Ann Arbor, MI 48109 USA; 2 Geriatrics and Extended Care Data and Analysis Center, VA Palo Alto Health Care System, 3801 Miranda Ave, Palo Alto, CA 94304 USA; 3 University of Michigan Medical School, 300 N. Ingalls Street, Ann Arbor, MI 48109 USA; 4 Sinclair School of Nursing and Department of Family and Community Medicine, University of Missouri, 915 Hitt Street, Columbia, MO 65211 USA

**Keywords:** Audit and feedback, Quality improvement, Life-sustaining treatments, User-centered design, Implementation, Tailoring feedback

## Abstract

**Background:**

The Long-Term Care QUERI program supported implementation of the Life-Sustaining Treatment Decisions Initiative in US Veterans Health Administration long-term care settings. The program worked with eleven Community Living Centers (CLCs) and twelve Home-Based Primary Care (HBPC) programs to increase rates of completed templates, using audit with feedback. We distributed monthly feedback reports to site champions showing the number of Veterans with appropriate documentation. Although feedback reports are a common implementation tool, little is known about the most effective ways to design, distribute, and support them. We sought to test tailoring reports with tips using site-specific data, as well as national comparator data.

**Methods:**

We conducted a cluster randomized controlled trial of monthly feedback reports utilizing site-tailored tips and national comparator data compared to our original feedback reports that included only graphical and numerical data. CLC and HBPC team members were invited to participate in brief surveys each quarter to determine if they had received and used the feedback reports. The outcome for CLC residents was the percent with a completed LST template any time prior to the 14th day of their stay. The outcome for HBPC residents was the percent of Veterans with a completed LST template by their second HBPC visit.

**Results:**

The response rate to the survey ranged between 6.8 and 19.3% of staff members across the CLC and HBPC sites with 12.8–25.5% of survey respondents reporting that they had seen the feedback reports. The linear regression models showed no significant association between receiving the enhanced feedback reports and having a higher documentation completion rate.

**Conclusions:**

Receiving feedback reports tailored to sites by including tips based on baseline context assessments and qualitative findings, and reports showing national comparator data, did not have an impact on the number of Veterans with a completed LST template. Having a higher proportion of CLC or HBPC team members view the reports was not associated with an increase in LST template completion. These findings suggest that tailored audit with feedback may not have been effective at the program level, although the proportion of respondents who reported seeing the reports was small.

**Supplementary Information:**

The online version contains supplementary material available at 10.1186/s43058-023-00510-7.

Contributions to the literature

- This cluster randomized controlled trial of tailored feedback reports using site-specific data is relatively novel and contributes to the literature on tailored implementation strategies in healthcare settings.
- Findings from this study are compromised by the lack of distribution of the feedback reports, which we learned by surveying respondents. Ascertaining whether feedback reports are received is rarely done in audit with feedback studies.
- Our findings highlight the importance of understanding the factors that influence staff access to feedback reports to make sure they are distributed and viewed more widely by staff to impact the desired behavior change.

## Background

The US Veterans Health Administration (VHA) National Center for Ethics in Health Care released the Life-Sustaining Treatment Decisions Initiative (LSTDI) in 2017, with a mandate for full implementation within 18 months of being released [1]. The LSTDI was created to ensure that Veterans’ goals, values, and preferences for life-sustaining treatments (LSTs) are elicited, documented in the electronic health record, and honored across the VA system. The associated life-sustaining treatment (LST) template and order set is the standardized, durable electronic health record tool used to document goals of care conversations, and orders generated through it are the only method of documenting LST decisions, such as whether the patient desires full cardiac and pulmonary resuscitation in case of arrest [2]. For inpatient settings, this requirement ensures the use of the template.

The Long-Term Care (LTC) Quality Enhancement Research Initiative (QUERI) program was funded in 2015 to support implementation of the LSTDI in VHA long-term care settings. LTC QUERI worked with VA-owned and operated nursing homes called Community Living Centers (CLCs) and Home-Based Primary Care programs (HBPC) across two VHA regional networks called Veterans Integrated Service Networks (VISNs) [3]. We identified site champions within each program who agreed to be the liaison for our work through contacting leadership in LTC at each facility in the two VISNs.

Our implementation strategy for the overall project was audit with feedback [3]. We selected it as our implementation strategy as it has been extensively studied as an approach to modifying behavior among specific groups of providers [4, 5]. Additionally, audit with feedback has been shown to improve the richness of physician documentation and our overall program goal was to support documentation of LST preferences in the electronic medical record [6, 7].

Audit with feedback involves aggregating clinical or other performance data, both over time and, in the case of unit or team feedback, over individual performance, and providing the aggregated data summary to individual practitioners, teams, or healthcare organizations. It has been shown to have a positive but modest absolute effect of increasing the likelihood of achieving a desired behavior change of about 4%, at the median [8]. Despite a strong body of literature, little is known about how to optimize the effectiveness of feedback interventions [9, 10]. Using comparator data and providing specific statements tailored to local conditions have been shown to improve the effectiveness of feedback interventions [8, 11]. Our initial feedback reports were developed through a user-centered design process [12].

Our objective in this sub-study was to test different approaches to tailoring feedback reports.

## Methods

In this paper, we report the results of a sub-study cluster randomized controlled trial of *tailored feedback reports* conducted as part of the Long-Term Care QUERI (LTCQ) program. The protocol papers for the LTCQ program were published previously [3, 13], and the main results for the Community Living Center (nursing home) component of the study were reported previously [14]. The main results for the Home-Based Primary Care component are currently under review.

### Overall study

**Fig. 1:**
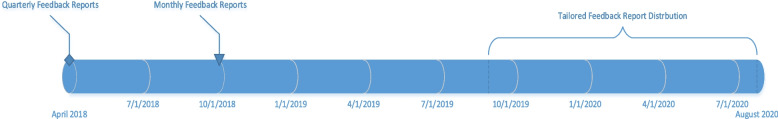
Timeline of feedback report distribution

The entire LTCQ program was conducted between October 2015 and August 2020. In April 2018, we started sending quarterly feedback reports to site champions that showed the number of Veterans who had a completed LST template while receiving care in a CLC or HBPC and increased the frequency to monthly in October 2018 (Fig. 1: Timeline of feedback report distribution). The feedback reports were sent by LTCQ staff to the site champion each month via email. The email included a reminder that the feedback reports could be shared with leadership and/or other team members. In these reports, no comparators or other information was provided. A total of eleven CLCs and twelve HBPC programs participated in the LTCQ program over the course of the entire study. Other details about the overall study are available through the previously published papers [3, 13].

### Intervention description for sub-study

We conducted a cluster randomized trial of two types of *tailored feedback reports* with mid-point cross-over between September 2019 and August 2020. In the first type, we provided tips for overcoming barriers and making use of facilitators identified through previous data collection (see the “Tailoring methods” section below). In the second type, we provided performance comparators based on national data for the type of program, either Community Living Centers (VA nursing homes) or Home-Based Primary Care. We provide an example of each type of tailored feedback report in Additional File 1. All sites participating in the LTCQ feedback report study were included in the randomized trial.

### Rationale

Our hypotheses were informed by the Clinical Performance Feedback Intervention Theory (CP-FIT) [15]. For the tip-tailored feedback reports, we hypothesized that feedback reports tailored with tips related to areas of organizational concern differ from non-tailored feedback reports in the following ways: greater distribution of the feedback report, more individuals reading the feedback report, more individuals expressing understanding of the report, more discussion of the report among providers, and, ultimately, increased proportion of Veterans with completed LST templates. We were attempting to increase *actionability* by providing additional prompts to the clinical champions and other team members to help them overcome identified barriers and use identified facilitators to increase the proportion of documented LST templates among eligible Veterans. We kept the tips short and succinct to limit the cognitive burden of reading and understanding the feedback reports.

For the comparator-tailored feedback reports, we hypothesized that feedback reports providing comparator data provide motivation to the individual or team receiving the report to improve performance if it is below the level of the comparison. Based on CP-FIT, an underlying mechanism is also *actionability* by communicating room for improvement as well as adding *social influence* by comparing to others in the same type of setting. The decision to include comparator data was motivated by the theory of planned behavior which emphasizes the potential for social pressure to influence changing professional practice [16].

We were interested in testing the effect of the tip-tailored reports compared to the comparator-tailored reports, and overall, the tailored reports to the non-tailored reports used throughout the study.

Feedback reports were sent by email from the point of contact (one individual) for each VISN in the research team to the champion at each site, and they were encouraged to share the reports with their respective care teams.

**Fig. 2:**
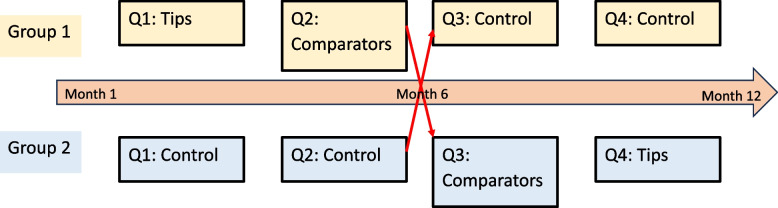
Trial Design

The trial lasted 12 months. The sites were divided into two groups as shown in Fig. 2. Group 1 received the tailored feedback reports (intervention) for the first 6 months, while Group 2 functioned as the control group receiving the non-tailored feedback reports (as initially designed for the full study). Within Group 1, each site received the tip-tailored reports for 3 months, and then the comparator-tailored reports for 3 months. We crossed over from intervention to control and vice versa at the mid-point of the trial. We used a crossover design because all sites were participating in the full LTCQ program, and we did not want to create an imbalance between sites. Our hypothesis was that the tailored feedback reports would result in an increase in the number of Veterans with documented LST templates. Our overall program goal was to support implementation of the LSTDI, and we felt it was important for all sites to receive the enhanced feedback reports. We exposed each site to the tip-enhanced reports followed by the comparator reports because we were interested in separating the effect of one type of enhancement from another, if our overall hypothesis that there was a difference between tailored and non-tailored feedback reports was supported.

### Tailoring methods

In one of the two VISNs involved in the sub-study, we asked CLC and HBPC team members to complete the Organizational Readiness to Change Assessment (ORCA) survey to serve as a baseline context assessment. In the other VISN, we conducted qualitative interviews based on the Consolidated Framework for Implementation Research (CFIR) for the baseline context assessment. In both cases, these were conducted over the first 18 months of the overall study. We used two different methods based on the strengths and preferences of the research team, which was geographically divided into two groups, one for each VISN.

The ORCA assesses respondents’ beliefs and attitudes about the strength of evidence for a specific evidence-based intervention (EBI), the favorability of the organizational context for change, and the capability to facilitate the implementation of the EBI based on individuals’ perceptions of factors within these domains [17, 18]. The ORCA scales and sub-scales are rated on a scale from 1 (strongly disagree) to 5 (strongly agree) [17, 18]. We analyzed these data descriptively for each site.

We conducted qualitative interviews with the site champions from the second VISN to assess barriers and facilitators to implementing the LSTDI, as well as gauge use and distribution of the feedback reports. We used the CFIR to code the interview data, assessing salient and frequent themes emerging from the interviews, coded into CFIR constructs. The CFIR is one of the most widely used determinant frameworks to assess and describe the context of potential barriers and facilitators to implementation [19–21]. CFIR constructs are rated for valence (+ / −) and strength [1, 2] with a + 2 indicating a strong influence on implementation, + 1 a weak to moderate influence on implementation, − 2 a strong negative influence on implementation, and − 1 a weak to moderate negative influence on implementation [22].

We modified the reports in September 2019 to include one version providing tips tailored (tip-tailored feedback reports) to each site based on data either from the ORCA surveys or CFIR interviews, and another version that provided LST template comparator data based on the entire VA (Additional File 1 – Feedback Report Examples). The LTCQ team along with a Co-Investigator who also served as a CLC Director were involved in the tailoring process.

Our feedback reports were tailored to sites by delivering key messages/tips focused on areas of organizational concern based on the baseline ORCA survey results and the qualitative interviews coded using the CFIR (see Additional File 2). Eleven CLC and HBPC programs from one VISN received key messages/tips based on baseline ORCA survey results and twelve CLC and HBPC programs from the other VISN received tips based on qualitative interviews coded using the CFIR. For each site, members of the research team (AES and JK) identified constructs with high vs. low ORCA scores and high vs. low CFIR ratings.

Each tip-tailored report included one positive tip related to areas of organizational concern in which the site scored highly, and one reinforcing tip related to areas of organizational concern in which site scores were lower. A total of seventeen tips were generated for the feedback reports for use across all sites. Each site received six positive and six reinforcing tips over the course of the trial. Positive tips included ORCA and CFIR constructs related to knowledge and beliefs about intervention/compatibility, strong leadership, strong staff culture, strong clinical champions, positive networks and communications, available resources, and self-efficacy. Reinforcing tips included ORCA and CFIR constructs related to lack of leadership support, lack of resources, lack of clinical champion self-efficacy, and lack of staff buy-in.

The comparator-tailored report included national comparator data on LST template completion rates. These reports included a comparator line on the bar chart that showed the median performance for all CLC or HBPC sites nationally across the VA in each month. As the LSTDI was still in the early implementation phase, we decided to use median performance data as the comparator so that lower-performing sites would not disengage from the feedback or view it as unattainable [23]. We did not modify the feedback report delivery mechanism or other components of the intervention during the trial.

### Evaluation methods

We used a randomized design with crossover controls to assess the effect of tailoring overall, and we planned to assess differences between tip and comparator tailoring if we found an overall difference between tailored and non-tailored reports.

Every 3 months during the trial we asked CLC and HBPC team members to complete a brief survey (the Feedback Uptake Scale [24]) reporting on receipt and understanding of the feedback report using REDCap. The data from this survey allowed us to assess some measures of implementation fidelity, including whether or not intended recipients (the care teams) received and understood the feedback. The survey asked staff: if they received the report, how much of the report they read, how well they felt they understood the information in the report, how useful they found the report, and whether they discussed the report with any other staff at their unit or facility [10].

Site champions and CLC and HBPC leadership determined who would be invited to participate in the survey by providing the LTCQ team with the list of staff email addresses to invite to complete the survey. Some sites wanted to include all CLC or HBPC team members while others only wanted to include team members who were involved in completing LST templates. The range of CLC team members invited to complete the survey was 13–107 with a median of 48 team members across the eleven programs. The range of HBPC team members invited to complete the survey was 12–60 with a median of 26 team members across the twelve programs.

For both the CLC and HBPC analyses, we aggregated and compared months between September 2019 and February 2020 with March 2020 and August 2020, using segmented regression analysis to conduct interrupted time series methods [25]. LST templates are stored as specific data elements, called LST health factors, in the VA’s Corporate Data Warehouse (CDW). Data for the completed templates from CDW were merged with census data for Veterans from the CLCs and HBPC programs participating in LTC QUERI. The data were aggregated using SAS version 9. The regression models were run in R version 4.0 using the linear model function.

**Fig. 3:**
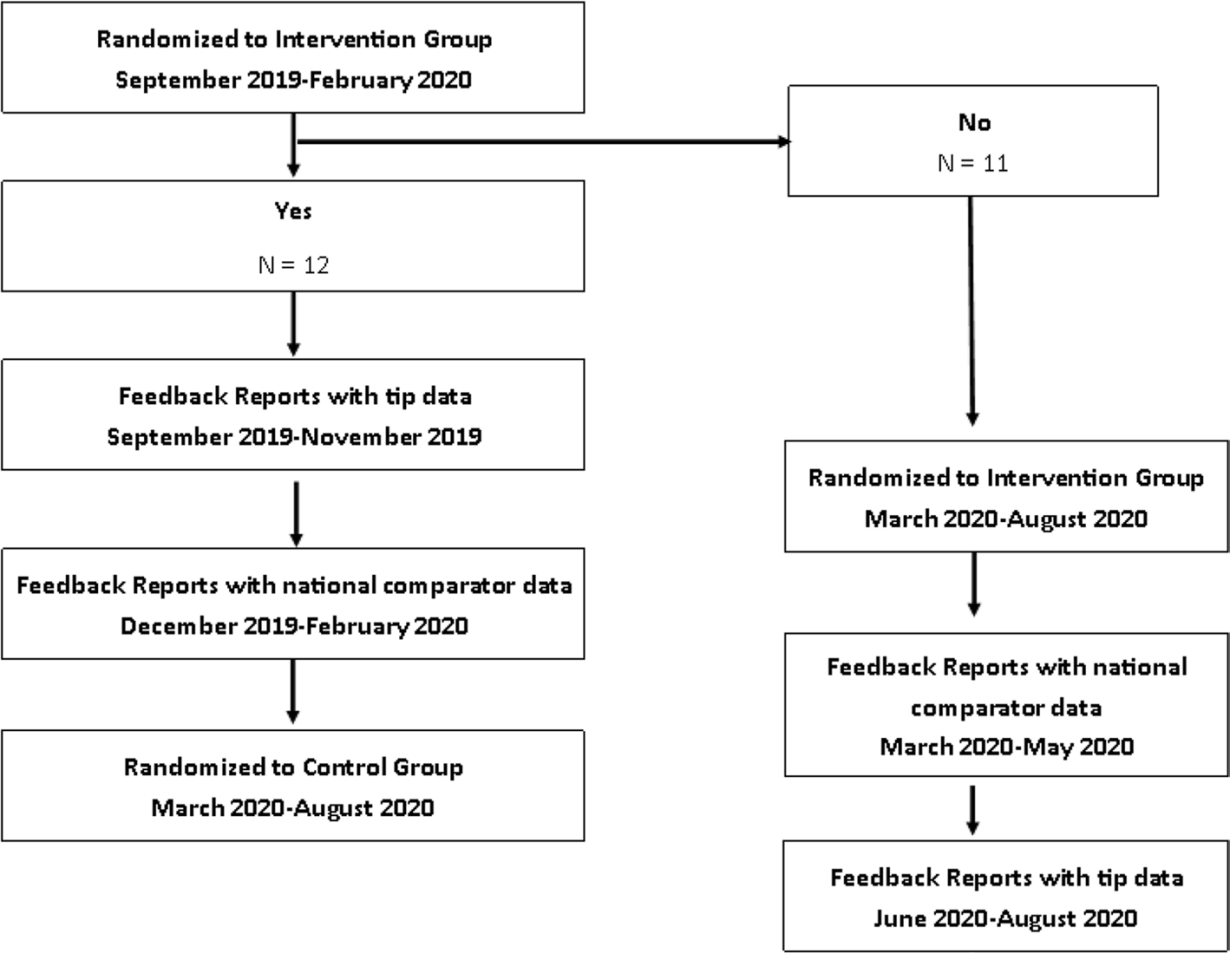
Consort Diagram of Intervention and Control Sites

We analyzed data from 11 CLCs; seven CLCs were in Group 1 and four were in Group 2 (Fig. 3). The percent of CLC residents with a complete LST template any time prior to the 14th day of the stay and prior to discharge was modeled as the outcome variable. We used 14 days as the timepoint because CLC residents would have multiple encounters with providers after they are admitted, providing numerous opportunities to complete the LST template.

We included data from 12 HBPC sites; five HBPC sites were in Group 1 and seven were in Group 2. For HBPC sites, the outcome modeled was the percent of Veterans with a completed LST template at any time until their second HBPC visit. The second visit was chosen as the outcome as the admission visit is often lengthy with numerous assessments, so the LST template may not be addressed until the second visit.

We used a linear model to estimate the effect of these independent variables—an indicator for being in the intervention group, an indicator for the second 6 months of the study, and an interaction term capturing the proportion of survey respondents who had seen the reports during the second 6 months of the study—for both the CLC and HBPC analyses, which were conducted separately.

## Results

Table 1 shows the average response rates to the Feedback Uptake Scale survey across each time interval and cluster. Overall, the response rates ranged between 6 and 19% across the CLC and HBPC sites.

**Table 1:** Response rates to quarterly surveys

Time interval	HBPC sites			CLC sites		
	***N* sites**	**Mean response rate**	**Range**	***N* sites**	**Mean response rate**	**Range**
1st 6 months						
Non-intervention cluster	7	9.9%	0.0–14.9%	4	10.4%	7.3–14.1%
Intervention cluster	5	19.3%	6.4–60.4%	7	6.8%	3.4–11.5%
2nd 6 months						
Non-intervention cluster	5	11.5%	4.5–20.8%	7	7.2%	1.5–14.6%
Intervention cluster	7	9.4%	2.5–17.6%	4	19.0%	6.4–37.3%

Table 2 shows the overall number of people at each site who responded to the surveys, the number and percent who saw the feedback reports, and the average proportion of Veterans who had complete LST templates. The overall number of respondents who reported that they had seen the feedback reports was between 12.8 and 25.5% for CLC and HBPC sites.

**Table 2:** Respondent reports of receiving feedback reports and proportion of Veterans with LST templates (percentages are based only on reports received)

Time interval	HBPC sites				CLC sites			
	***N* sites**	***N* responses**	***N* received report (%)**	**Mean percent with LST template during 1st|2nd visit, or prior to admission**	***N* sites**	***N* responses**	***N* received report (%)**	**Mean percent with LST template—any time up to the first 14 days, prior to discharge**
1st 6 months								
Non-intervention cluster	7	47	6 (12.8%)	23.79%	4	53	11 (20.1%)	95.68%
Intervention cluster	5	51	13 (25.5%)	44.94%	7	47	10 (21.3%)	60.93%
2nd 6 months								
Non-intervention cluster	5	37	7 (18.9%)	53.65%	7	56	13 (22.7%)	81.08%
Intervention cluster	7	39	5 (12.8%)	44.40%	4	94	12 (13.3%)	95.08%

**Fig. 4:**
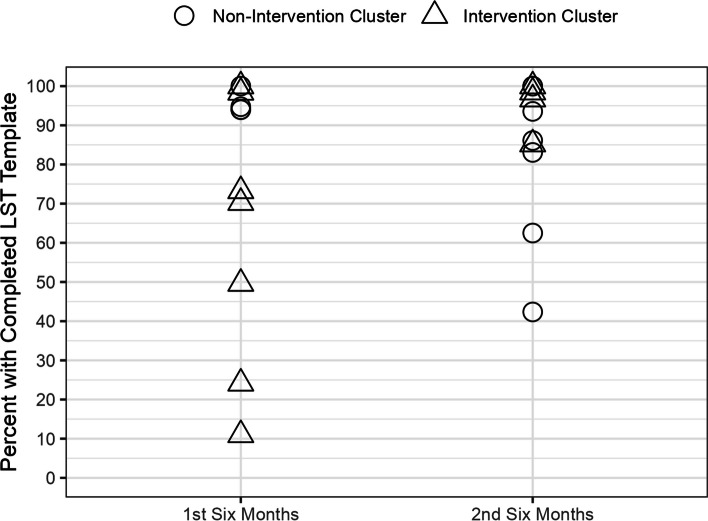
CLC Veterans with Completed LST Templates by Intervention and Control Groups

**Fig. 5:**
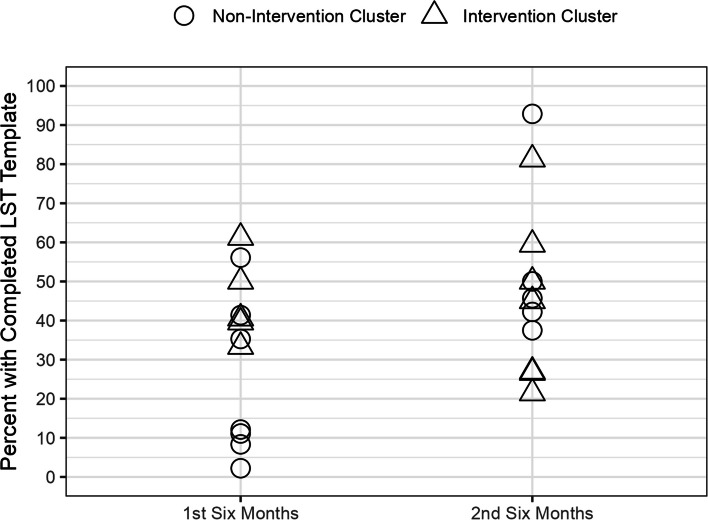
HBPC Veterans with Completed LST Templates by Intervention and Control Groups

Figures 4 (CLC Sites) and 5 (HBPC Sites) show the distribution of Veterans with completed LST templates in each 6-month interval in the control and intervention sites group. There was a wide distribution in the completion rate of LST templates across the 11 CLC and 12 HBPC sites. Overall, the proportions of completed LST templates were higher in the second 6-month interval with no significant difference between the intervention and control groups Fig. 5.

The linear regression models for both the CLC and HBPC sites modeled the respective outcomes with an indicator for being in the intervention group in the given 6-month interval, an indicator for the second 6 months of the study, and an interaction term capturing the proportion of survey respondents who had seen the reports during the second 6 months of the study; sites in the intervention group in one 6-month interval were in the non-intervention group in the other 6-month interval. Table 3 shows the results for the 2 linear regression models. The models showed no significant association between receiving the tailored feedback reports in a given 6-month interval and having a higher proportion of residents or Veterans with a completed LST templates. There was also no significant association between having a higher proportion with LST templates and the 6-month interval, although both models showed an increase in the proportion for the second 6-month interval. The interaction term for the proportion of survey respondents who had seen the reports in the second 6-month interval was also not significant. Because we found no significant difference overall between the tailored feedback reports and the non-tailored reports, we did not analyze the differences between the two types of tailored reports.

**Table 3:** Linear regression model results

Coefficients	**HBPC**			**CLC**		
	Estimate	Standard error	*P*-value	Estimate	Standard error	*P*-value
Intercept	30.687	7.048	0.000307	80.142	11.077	< 0.0001
Intervention group indicator	4.599	8.77	0.605727	-10.329	11.891	0.396
Six-month interval indicator	19.611	10.219	0.069361	5.939	15.069	0.698
Interaction	-48.873	53.823	0.37467	20.525	49.554	0.684

## Discussion

The results of the regression models indicate that neither the enhanced feedback reports nor having a higher proportion of survey respondents viewing reports was associated with any increase in the proportion of Veterans served having completed LST templates. While not statistically significant, it appears that the passage of time increased the proportion of eligible Veterans with completed LST templates, which likely reflects the overall success of the LSTDI implementation at the end of the intervention period. Prior research has found that it takes time to change practice, so if we had has a longer observation period, it may have resulted in a larger impact of the tailored feedback reports on completed LST templates in the targeted CLCs and HBPCs [26]. As we note in our Limitations and Challenges section, we were extremely time constrained.

Prior research has found that feedback is most effective when it is delivered by a supervisor or respected colleague, which is why we identified site champions from each program to be the liaison for the feedback reports [27]. However, we found that the feedback reports were rarely distributed widely by the site champion. From the survey, in general, site champions did not spend a great deal of time with the feedback reports and most did not distribute them to team members or others in the programs. We cannot tell from the surveys why this was the case, but it clearly affected the probability that any action would be taken based on the feedback reports. In some interviews done as part of the larger study, we learned that some site champions were reluctant to share the feedback reports out of concern that they might de-motivate staff if their performance appeared poor. This is in line with prior research that has found feedback perceived to be punitive is often less effective [28]. Others were concerned about taking staff time to review feedback reports given the pressure of their work in general.

We recommend that future projects that plan to utilize site champions to distribute feedback reports assess the site champion’s quality improvement capabilities and beliefs about data, which prior work has found influences engagement with feedback interventions [29]. Though the LTCQ team had been working with the site champions for close to 2 years at the time of this RCT, more time building relationships with the site champions may have mitigated skepticism and enhanced feedback acceptability [30]. The feedback reports were delivered to site champions via email and if we had also provided the opportunity for site champions to discuss the data with LTCQ staff, it may have supported the use of the feedback reports [31]. In our analysis of the overall project findings, a more intensive intervention which included coaching and frequent engagement with site champions and other staff was found to be more effective in increasing the proportion of eligible Veterans with completed LST templates [14].

## Challenges and limitations

There are some limitations to this work. Only a small number of CLCs and HBPC sites out of the close to 100 VA CLCS and several hundred HBPC teams nationally were included, and a lack of power may have impacted the ability to identify an effect of the tailored feedback reports. The characteristics of different VAMC sites vary significantly. We only looked at data across 1 year of time that began 2 years after the LTCQ team began sending feedback reports to sites; the feedback reports may have played a larger role in encouraging the use of the LST template in these earlier years. As more Veterans have a completed LST template, there is a declining proportion of the population that needs to be reached, especially in CLCs [14]. We note that the crossover design may have led to “contamination” between control and intervention sites. It is possible that the effects of the enhanced feedback reports received by the first intervention group could have affected the control period if the feedback reports were of more interest to staff after the tailoring period [32]. Based on the limited survey data that showed the reports were not distributed widely to staff, we do not feel this was a factor; we did not see much evidence that staff had access to the reports.

Furthermore, our study time frame was interrupted by the COVID-19 pandemic, which took attention and resources away from tasks such as LST template completion. During this time, many VA CLCs had to change their function to COVID units and/or had many staff members detailed to inpatient COVID units, so CLC staffing levels decreased. HBPC teams were affected by staffing shortages as well as restrictions on in-home visits. We can assume that these disruptions led to a decreased emphasis on the LSTDI and LST template completion. Another limitation of note is how few CLC and HBPC team members completed the quarterly surveys. We can hypothesize that a non-response is indicative of team members not receiving the feedback reports from their site champion.

Our findings with respect to whether the feedback reports actually reached the intended recipients—the care teams providing direct care to Veterans in the two settings—are an important issue of concern. In many reports of feedback intervention studies, there is no discussion of whether or how assessment was made of reports reaching their intended recipients. It is difficult to construct a cogent theory of how feedback can work if the intended recipients do not receive it. Ours is one of a small number of studies of feedback interventions that did attempt to measure receipt of the feedback report, as well as whether it was understood. We suggest that this is an important gap in the audit with feedback literature that should be addressed in future studies.

## Conclusions

Our randomized controlled trial of tailored audit with feedback showed that tailored feedback reports did not significantly enhance processes or outcomes compared to simple feedback reports. This was in the context of poor response to surveys assessing whether staff received the feedback reports; when surveys were completed, they showed that it was rare that staff had received the feedback report. We hypothesized that reports tailored to areas of site-specific organizational concern and those that provided comparator data would be distributed more widely to CLC and HBPC staff members than non-tailored feedback reports. The tailored reports did not lead to an increased proportion of Veterans with completed LST templates during the study period. Future research and implementation science teams should consider how to support and encourage greater distribution of tailored audit with feedback to result in the desired behavior change. Measuring whether feedback reports reach their intended audience is critical.

### Supplementary Information

**Additional file 1.** Feedback Report Examples.

**Additional file 2.** Tips for Tip-Tailored Feedback Reports.

## Data Availability

Data will be available from the authors on request.

## Abbreviations

CFIR: Consolidated Framework for Implementation Research
CLC: Community Living Centers
EBI: Evidence-Based Intervention
HBPC: Home-Based Primary Care
LST: Life-sustaining treatments
LSTDI: Life-Sustaining Treatment Decisions Initiative
LTC: Long-term care
LTCQ: Long-Term Care QUERI
ORCA: Organizational Readiness to Change Assessment
QUERI: Quality Enhancement Research Initiative
VISN: Veterans Integrated Service Networks
