# Supplementary material for: Randomized controlled trial of tailored audit with feedback in VHA long-term care settings
Source: Implement Sci Commun. 2023 Oct 26;4:129. doi: 10.1186/s43058-023-00510-7 (PMC10601134; doi:10.1186/s43058-023-00510-7)

To: VAMC CLC Providers and Staff

From: Long-Term Care QUERI program

Subject: Feedback Report on Documented Life-Sustaining Treatment Decisions in CLC

Date: May 4, 2020

The following pages present information about the number of completed Life Sustaining Treatment (LST) templates for Veterans at the VAMC CLC.

Please review these charts to encourage reflection on your current practice and how your CLC might increase the number of goals of care conversations (GoCCs) and completed LST order templates.

To better understand the data, please keep in mind these points:

- The report includes data only for Veterans who were newly admitted to your CLC in the last 6 months, month by month
- Data come from the LST template in CPRS. Only templates with all 4 required elements completed were counted
- If a Veteran had more than one LST template completed in a month, only the first template was counted
- Each Veteran was counted only once even if they had multiple stays.

More information about the goals and implementation of the LSTDI, including a step-by-step implementation guide, is available at the following website: `http://vaww.ethics.va.gov/LST.asp`.

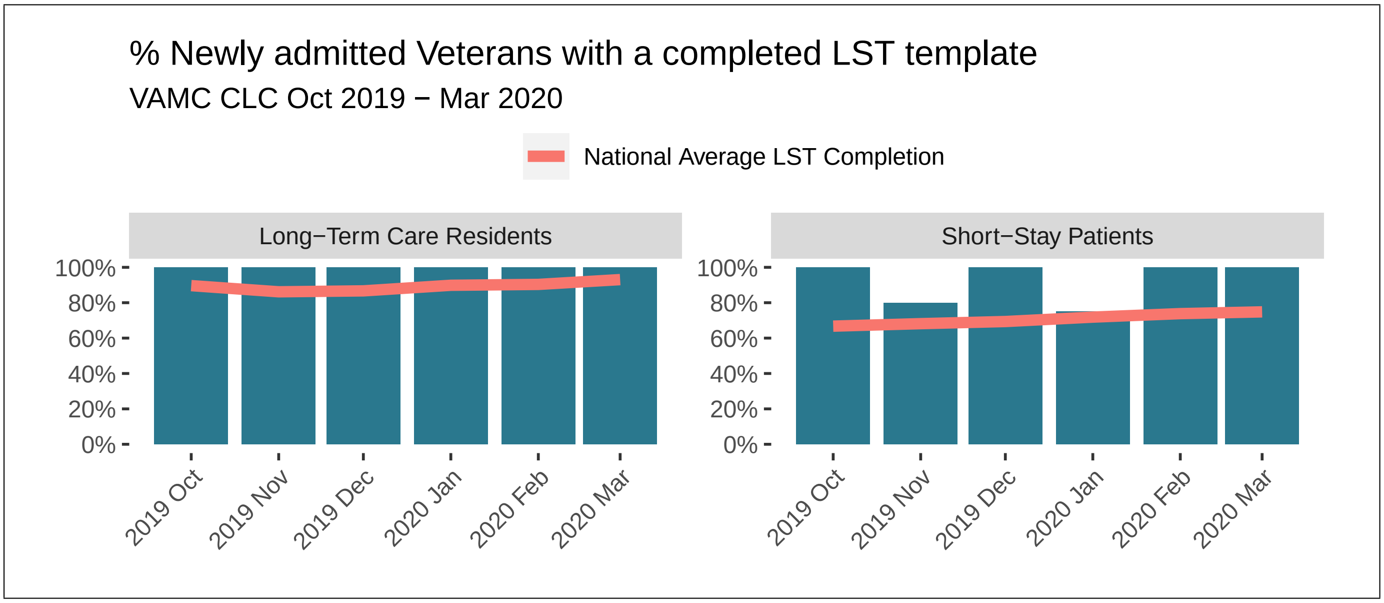

If you have any questions about this report, you can contact:

Jennifer Kononowech, Project Coordinator (Jennifer.Kononowechva.gov)

Thank you for your efforts to conduct GoCCs and complete LST templates with Veterans and their families to improve care and the quality of life for our Veterans.

To: HBPC Providers and Staff

From: Long-Term Care QUERI program

Subject: Feedback Report on Documented Life-Sustaining Treatment Decisions in HBPC

Date: September 6, 2019

The following pages present information about the number of completed Life Sustaining Treatment (LST) templates for Veterans in the HBPC program.

Please review these charts to encourage reflection on your current practice and how your HBPC program might increase the number of goals of care conversations (GoCCs) and completed LST order templates.

To better understand the data, please keep in mind these points:

- The report includes data only for Veterans who were newly admitted to your HBPC in the last 6 months, month by month
- Data come from the LST template in CPRS. Only templates with all 4 required elements completed were counted
- If a Veteran had more than one LST template completed in a month, only the first template was counted
- Each Veteran was counted only once even if they had multiple admissions.

More information about the goals and implementation of the LSTDI, including a step-by-step implementation guide, is available at the following website: `http://vaww.ethics.va.gov/LST.asp`.

Tips based on an assessment of your HBPC:

- Staff at your site value using goals of care conversations, and can be supported by emphasizing the common goals you share, including the value of understanding Veteran preferences and goals.
- Perceiving that there aren't enough resources to do specific work is often related to how high a priority it is. Two ways to try to increase the priority level of an initiative like the LSTDI are to show data about how your facility is doing, and to tell stories about how important this is to individual Veterans.

If you have any questions about this report, you can contact:

- Jennifer Henry, Project Coordinator (Jennifer.Henry2@va.gov)

Thank you for your efforts to conduct GoCCs and complete LST templates with Veterans and their families to improve care and the quality of life for our Veterans.

How many total newly admitted Veterans have a completed LST template?

HBPC Feb 2019 − Jul 2019

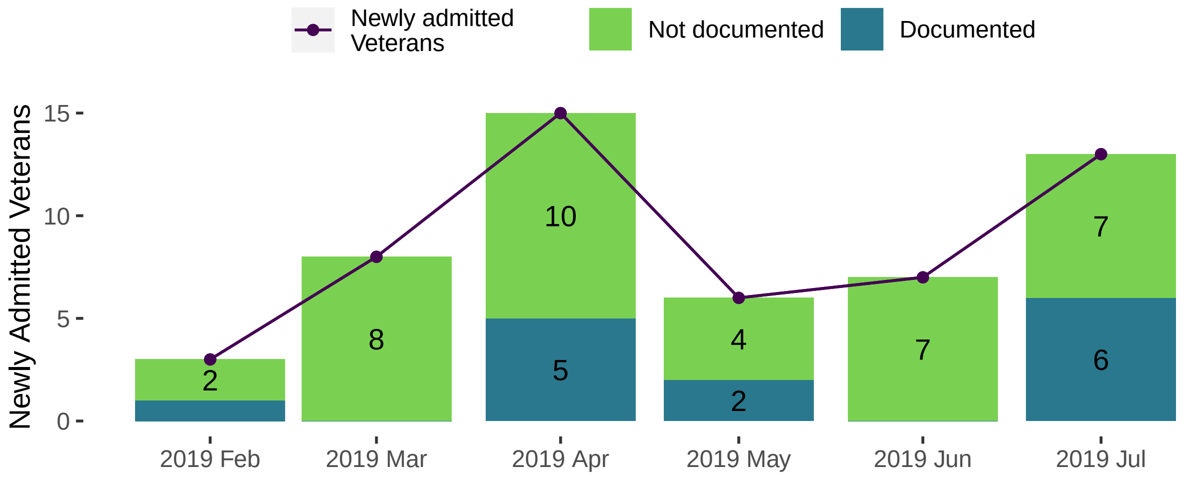

During which visit were LST templates completed?

HBPC Feb 2019 − Jul 2019

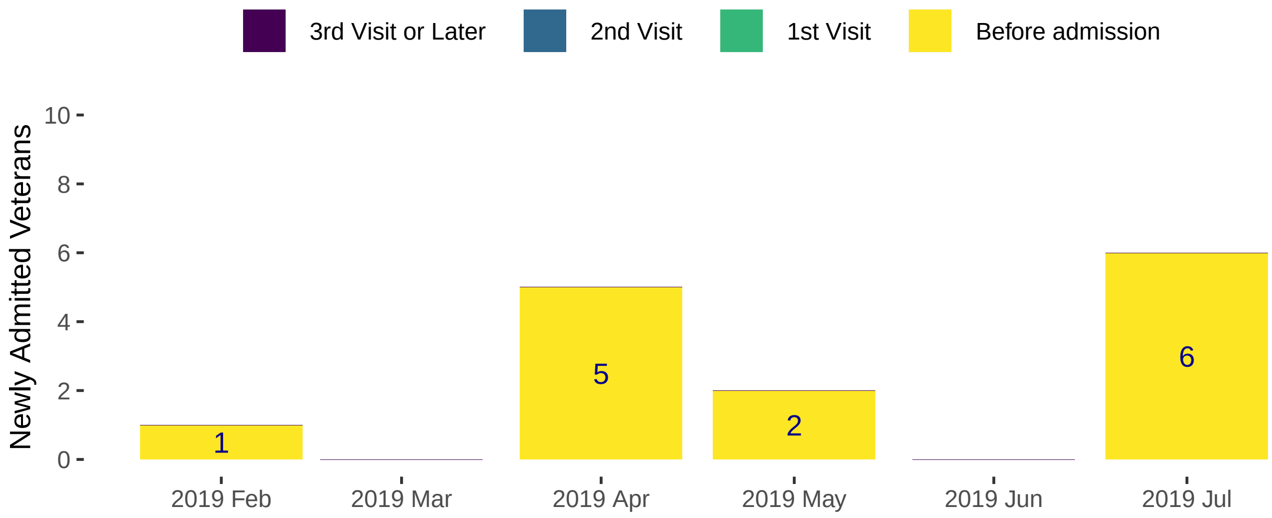

Supplement: Supplementary file 1 — Additional file 1. Feedback Report Examples. [file 43058_2023_510_MOESM1_ESM.pdf]
