# Supplementary material for: Randomized controlled trial of tailored audit with feedback in VHA long-term care settings
Source: Implement Sci Commun. 2023 Oct 26;4:129. doi: 10.1186/s43058-023-00510-7 (PMC10601134; doi:10.1186/s43058-023-00510-7)
Supplement: Supplementary file 2 — Additional file 2. Tips for Tip-Tailored Feedback Reports. [file 43058_2023_510_MOESM2_ESM.docx]

Tips for enhanced feedback reports

1. Positive tips related to areas in which sites score highly:
   1. Strong on evidence and values consistency/knowledge and beliefs about intervention/compatibility
      1. Staff at your site value using goals of care conversations and can be supported by emphasizing the common goals you share, including the value of understanding Veteran preferences and goals.
      2. Staff value the evidence underlying the Life Sustaining Treatment Decision Initiative, and they are acting on that evidence, which is great!
   2. Strong on leadership
      1. Staff perceive that leaders at your site support their work to fulfill the Life Sustaining Treatment Decision Initiative, and value their efforts—saying thank you goes a long way!
   3. Strong on staff culture
      1. Staff perceive that their peers are strongly supportive of the LSTDI, and of their efforts. This is an important thing to celebrate!
   4. Strong on clinical champion
      1. Staff perceive that the Life Sustaining Treatment Decisions Initiative clinical champion at your site has been instrumental in the success of our implementation, which is great!
   5. Networks and communications
      1. Staff perceive that communication at your facility regarding the LSTDI is positive. This is an important thing to celebrate!
      2. Staff at your site are happy with the communication received regarding the LSTDI, which is great!
   6. Available resources
      1. Staff at your site perceive that they have adequate resources to support the implementation of the LSTDI, which is great!
   7. Self-efficacy
      1. Staff at your site report high levels of self-efficacy, which means they believe in their ability to achieve implementation goals, such as the LSTDI. This is an important thing to celebrate!
      2. Staff perceiving that they can successfully implement new initiatives, such as the LSTDI, often leads to a successful implementation!
2. Reinforcing tips related to areas in which sites do not score highly:
   1. Lack of leadership support
      1. Leadership support can seem less than enthusiastic for a new initiative like the LSTDI. This may be because it seems like a lower priority than other initiatives; offering comparative data to show how you’re doing compared to facilities like yours can help to establish this initiative as a priority.
      2. Leaders can get very immersed in management issues. Sometimes telling them a story about how valuable a goals of care conversation is for a specific Veteran can help them understand the importance of this work.
   2. Lack of resources
      1. Perceiving that there aren’t enough resources to do specific work is often related to how high a priority it is. Two ways to try to increase the priority level of an initiative like the LSTDI are to show data about how your facility is doing, and to tell stories about how important this is to individual Veterans.
      2. The number of initiatives in the VA can be overwhelming when you don’t feel there are enough resources to support it. Staff at your site strongly support the evidence of the LSTDI. Remember this support when you’re facing resource constraints.
      3. Implementing a new national initiative in the VA can be time and resource consuming. Talk to a Veteran about how important the LSTDI is to them to remind yourself of why you’re doing this work.
   3. Clinical champion lack of self-efficacy
      1. Finding a role model, perhaps at another facility, can help overcome feelings of self-doubt about leading an initiative such as the LSTDI. There are several resources for finding a role model, including contacting staff at the VA National Center for Ethics in Health Care who support the LSTDI.
   4. Lack of buy-in from staff/culture
      1. Staff may not perceive the importance of having goals of care conversations with Veterans, or the importance of the LSTDI as a whole. One way to get more buy-in is to share the data about how well the teams are doing in terms of having the conversations, and sharing stories of how important this can be for Veterans.
